# Exposure to COVID-19 patients increases physician trainee stress and burnout

**DOI:** 10.1371/journal.pone.0237301

**Published:** 2020-08-06

**Authors:** Thomas G. Kannampallil, Charles W. Goss, Bradley A. Evanoff, Jaime R. Strickland, Rebecca P. McAlister, Jennifer Duncan

**Affiliations:** 1 Department of Anesthesiology, Washington University School of Medicine, Saint Louis, Missouri, United States of America; 2 Institute for Informatics, Washington University School of Medicine, Saint Louis, Missouri, United States of America; 3 Division of Biostatistics, Washington University School of Medicine, Saint Louis, Missouri, United States of America; 4 Department of Medicine, Washington University School of Medicine, Saint Louis, Missouri, United States of America; 5 Department of Obstetrics & Gynecology, Washington University School of Medicine, Saint Louis, Missouri, United States of America; 6 Department of Pediatrics, Washington University School of Medicine, Saint Louis, Missouri, United States of America; Fukushima Medical University School of Medicine, JAPAN

## Abstract

The coronavirus disease 2019 (COVID-19) pandemic has put considerable physical and emotional strain on frontline healthcare workers. Among frontline healthcare workers, physician trainees represent a unique group—functioning simultaneously as both learners and caregivers and experiencing considerable challenges during the pandemic. However, we have a limited understanding regarding the emotional effects and vulnerability experienced by trainees during the pandemic. We investigated the effects of trainee exposure to patients being tested for COVID-19 on their depression, anxiety, stress, burnout and professional fulfillment. All physician trainees at an academic medical center (*n* = 1375) were invited to participate in an online survey. We compared the measures of depression, anxiety, stress, burnout and professional fulfillment among trainees who were exposed to patients being tested for COVID-19 and those that were not, using univariable and multivariable models. We also evaluated perceived life stressors such as childcare, home schooling, personal finances and work-family balance among both groups. 393 trainees completed the survey (29% response rate). Compared to the non-exposed group, the exposed group had a higher prevalence of stress (29.4% vs. 18.9%), and burnout (46.3% vs. 33.7%). The exposed group also experienced moderate to extremely high perceived stress regarding childcare and had a lower work-family balance. Multivariable models indicated that trainees who were exposed to COVID-19 patients reported significantly higher stress (10.96 [95% CI, 9.65 to 12.46] vs 8.44 [95% CI, 7.3 to 9.76]; *P* = 0.043) and were more likely to be burned out (1.31 [95% CI, 1.21 to1.41] vs 1.07 [95% CI, 0.96 to 1.19]; *P* = 0.002]. We also found that female trainees were more likely to be stressed (*P* = 0.043); while unmarried trainees were more likely to be depressed (*P* = 0.009), and marginally more likely to have anxiety (*P* = 0.051). To address these challenges, wellness programs should focus on sustaining current programs, develop new and targeted mental health resources that are widely accessible and devise strategies for creating awareness regarding these resources.

## Introduction

The rapid spread of the coronavirus disease 2019 (COVID-19) pandemic has put considerable strain on the physical, social, economic, and mental well-being of nearly the entire population of the United States. If previous epidemics such as the SARS (Severe Acute Respiratory Syndrome) and Ebola outbreaks are an indication, frontline healthcare workers are negatively impacted with an immediate psychological and occupational burden including health fear, loneliness, anxiety, and insomnia [1–3]. This is because frontline healthcare workers face a greater risk of exposure, greater workload, moral dilemmas during care, and have to deal with a continuously changing clinical practice environment (e.g., telemedicine replacing face-to-face encounters, change of evidence-base for care) [4]. Systematic reviews have suggested that risk factors contributing to poor mental health outcomes for healthcare workers include their level of exposure to the disease, being quarantined, and personal health fears [5]. These concerns were reflected in recent studies with healthcare workers exposed to patients with COVID-19 in China, who had a high prevalence of mental health symptoms including depression, anxiety, insomnia and distress [6, 7].

Much of the research on wellness during the COVID-19 pandemic has focused on nurses, attending physicians and healthcare support staff [6, 8], and have not included physician trainees. Physician trainees represent a unique group, as they function simultaneously as both learners and caregivers, who often have less autonomy and control in their work-setting. Although there is a limited understanding regarding the emotional effects and vulnerability experienced by trainees at the frontlines of COVID-19 patient care, recent anecdotal reports have described concerns about their safety, safety of their patients, and the implications of their decisions on their family [9]. There is also very preliminary evidence that trainees in the frontline of care—either testing or caring for patients—are being infected with COVID-19 themselves, with these numbers being higher in infection hotspots [10].

Even prior to the COVID-19 pandemic, depression, distress, and burnout were higher among physician trainees as compared to the general US working population. [11, 12] As such, understanding the impact of the pandemic on trainees and developing appropriate strategies to address the strains on the trainee workforce is of paramount importance [13]. The purpose of this study is to investigate the effect of physician trainees’ exposure to COVID-19 patients in their clinical roles on their mental health and wellness outcomes. We hypothesize that physician trainees’ exposure to COVID-19 patients are likely to be associated with poorer mental health outcomes and increased burnout.

## Method

### Participants

We conducted a web-based survey of all physician trainees (residents and clinical fellows) at Washington University School of Medicine, Barnes Jewish Hospital and St Louis Children’s Hospital. Email invitations with a link to a voluntary, de-identified survey was sent to 1375 physician trainees on April 10, 2020. A reminder was sent on April 17, 2020 and the survey was closed on April 25, 2020. Survey respondents were offered the opportunity to participate in a gift card raffle for participation. The study was approved by the institutional review board of Washington University. Prior to completing the survey, all participants read an “information sheet” that included details of the study; by completing the survey, participants provided consent to participate in this research study (IRB #202004021, Washington University).

### Survey

The survey included questions from several domains. Demographic and training program details included race, sex, marital status (married, domestic partner, single), occupation of significant other (if relevant), clinical role (resident, fellow), residency or fellowship program, and year in program. Additional questions on current clinical responsibilities (clinical services with and without patient contact, education, research, and quarantined) were also included.

We also asked four questions regarding perceived life stressors including financial concerns, childcare and home schooling (if relevant) and care of elder relatives (if relevant). These questions were asked in the format, “Currently how stressed are you about…,” with responses on a 5-point scale ranging from “not at all” to “extremely” stressed. These questions were developed based on informal conversations with trainees regarding the potential stressors that they faced during the pandemic.

Additional questions related to the work-family balance and awareness of institutional services were also included. Work-life balance questions were derived from the National Institute of Occupational Health and Safety (NIOSH) [14] focusing on the demands of the respondents’ work on their family life and the ability to take time off from work. The following questions were included: how often does the demands of your job interfere with your family life; how often does the demands of your family interfere with your work on the job (both questions rated on a scale from “often” to “never”); and how hard is to take time off during your work to take care of personal or family matters (rated on a scale from “not at all hard” to “very hard”). Finally, we also included questions regarding the usage of various institutional (hospital and school of medicine) and graduate medical education (GME) wellness resources among the participants. These questions asked respondents to indicate their usage (use, did not use, did not use/not aware) of wellness programs, COVID-19 wellness support resources, housing, and childcare resources.

Depression, anxiety and stress were ascertained using the short-form depression DASS-21 (Depression Anxiety Stress Scale) [15]; DASS-21 is a validated twenty-one item instrument that is correlated with measures of depression, anxiety and stress and has previously been used widely with the adult population [16, 17]; it has also been used with the physician trainee population [18]. Its reliability has been replicated among clinical and non-clinical samples [19, 20] and has been shown to be concurrently valid with other scales of depression, stress and anxiety including the Beck Depression Inventory, the Beck Anxiety Inventory and the State-Trait Anxiety Inventory Trait [21].

Burnout and professional fulfillment were assessed using the Stanford Professional Fulfillment Index (PFI). PFI is a 16-item survey that combines burnout—based on workload exhaustion and interpersonal disengagement (depersonalization)—and professional fulfillment [22]. The burnout components of the PFI correlates with the commonly used Maslach Burnout Inventory on the emotional exhaustion and depersonalization scales; the professional fulfillment is correlated with “quality of life” [22, 23]. PFI also has an advantage over similar scales as the inventory questions are aligned towards capturing burnout and professional fulfillment in the “past two weeks.”

### Exposure

The primary exposure variable was the response to the question “in your current clinical role are you caring for patients currently being tested for COVID-19” with a response choice of “Yes/No.”

### Outcomes

We considered five outcomes: depression, anxiety, stress (as measured using the DASS-21), burnout and professional fulfillment (as measured using the PFI).

### Statistical analysis

Race was categorized as Caucasian or non-Caucasian, sex was categorized as female or not female, and marital status was categorized as married or not married. Comparisons of variables between the exposed and unexposed groups were assessed using chi-square tests or Fisher’s exact tests, as appropriate.

For simple comparisons between the exposure groups, outcomes from the DASS-21 were categorized as normal or non-normal as follows: Depression (0–9 normal, 10 or greater non-normal), anxiety (0–7 normal, 8 or greater non-normal), and stress (0–14 normal, 15 or greater non-normal). These cut-points were based on previously published literature on the DASS-21 scale [17]. Burnout was determined from the average item score for the workload and depersonalization scales (score range 0 to 4), using a cut-point of 1.33 as described in Trockel et al (2018) [22], where scores greater than or equal to 1.33 was considered as “burned out.” Similarly, for professional fulfillment, an item score of greater than or equal to 3.0 was used as the cut-point (scale range 0–4), which has been shown to be correlated with physicians indicating their quality of life as being “very good.”[22].

For univariable and multivariable analyses, professional fulfillment and burnout outcomes were analyzed as continuous outcome variables, and outcomes from the DASS-21 (depression, anxiety and stress) were analyzed as count outcome variables. Associations between the exposure groups and professional fulfillment and burnout outcomes were analyzed using linear least-squares regression analyses; depression, anxiety and stress outcomes were analyzed using negative binomial regression analyses with a log link function. Exposure effects were adjusted in multivariable models by including covariates with *P*-values < 0.10 in the univariable analyses. *P*-values < 0.05 were considered significant, unless otherwise mentioned. All analyses were conducted using SAS version 9.4 (SAS Institute Inc, Cary, NC, USA).

## Results

There were 403 responses to the survey. After removing 10 duplicate entries, there were a total of 393 completed surveys (~29% response).

### General characteristics

Participants were primarily residents (66%), women (55%), Caucasian (63%) and were married (62%). Nearly 80% of the respondents were in the first three years of their training. 55% of the participants were exposed to patients who were being tested for COVID-19. 16% of the respondents had no encounters that required direct patient interactions and 12% had an emergency room or intensive care-based clinical activity.

Compared to the non-exposed group, the exposed group experienced moderate to extremely high perceived stress regarding childcare (61.7% vs. 39.2%, *P* = 0.026) and reported considerably lower work-family balance, including that their job duties interfered with their family life (sometimes or often, 68.2% vs. 55.4%, *P* = 0.009) and more difficulty in taking time off for attending to personal or family matters (somewhat or very hard, 74.1% vs. 47.7%, *P*<0.0001). There were no statistically significant differences between the groups for other perceived stressors such as homeschooling, personal finances or elder relative care (see Table 1).

**Table 1:** Summary descriptive table of the considered variables in the survey separated into trainees exposed to patients being tested for COVID-19 and trainees not exposed to such patients.

Variable label	Group	All Trainees	Trainees exposed to COVID-19 testing	Trainees NOT exposed to COVID-19 testing	*P*-value
Clinical role	Fellow	132/393 (33.6%)	67/218 (30.7%)	65/175 (37.1%)	0.18
	Resident	261/393 (66.4%)	151/218 (69.3%)	110/175 (62.9%)	
Female	No	175/393 (44.5%)	106/218 (48.6%)	69/175 (39.4%)	0.068
	Yes	218/393 (55.5%)	112/218 (51.4%)	106/175 (60.6%)	
Caucasian	No	147/393 (37.4%)	79/218 (36.2%)	68/175 (38.9%)	0.59
	Yes	246/393 (62.6%)	139/218 (63.8%)	107/175 (61.1%)	
Married	No	175/393 (44.5%)	105/218 (48.2%)	70/175 (40%)	0.11
	Yes	218/393 (55.5%)	113/218 (51.8%)	105/175 (60%)	
Children at home	No	295/393 (75.1%)	171/218 (78.4%)	124/175 (70.9%)	0.084
	Yes	98/393 (24.9%)	47/218 (21.6%)	51/175 (29.1%)	
> = 4 years on program	No	311/387 (80.4%)	166/213 (77.9%)	145/174 (83.3%)	0.18
	Yes	76/387 (19.6%)	47/213 (22.1%)	29/174 (16.7%)	
Stressed about home schooling?	Not at all, Little	70/98 (71.4%)	36/47 (76.6%)	34/51 (66.7%)	0.28
	Somewhat, Quite a bit, Extremely	28/98 (28.6%)	11/47 (23.4%)	17/51 (33.3%)	
Stressed about childcare?	Not at all, Little	49/98 (50%)	18/47 (38.3%)	31/51 (60.8%)	**0.026**
	Somewhat, Quite a bit, Extremely	49/98 (50%)	29/47 (61.7%)	20/51 (39.2%)	
Stressed about personal finances?	Not at all, Little	265/393 (67.4%)	147/218 (67.4%)	118/175 (67.4%)	0.99
	Somewhat, Quite a bit, Extremely	128/393 (32.6%)	71/218 (32.6%)	57/175 (32.6%)	
Stressed about care for your relatives?	Not at all, Little	3/17 (17.6%)	2/7 (28.6%)	1/10 (10%)	0.54†
	Somewhat, Quite a bit, Extremely	14/17 (82.4%)	5/7 (71.4%)	9/10 (90%)	
How often do the demands of your job interfere with your family life?	Never, Rarely	147/392 (37.5%)	69/217 (31.8%)	78/175 (44.6%)	**0.0094**
	Sometimes, Often	245/392 (62.5%)	148/217 (68.2%)	97/175 (55.4%)	
How hard is it to take time off during your work to take care of personal or family matters?	Not at all hard, Not too hard	147/390 (37.7%)	56/216 (25.9%)	91/174 (52.3%)	< .0001
	Somewhat hard, Very hard	243/390 (62.3%)	160/216 (74.1%)	83/174 (47.7%)	
Depression	Not Normal (Mild to Extremely Severe)	107/393 (27.2%)	61/218 (28%)	46/175 (26.3%)	0.71
	Normal	286/393 (72.8%)	157/218 (72%)	129/175 (73.7%)	
Anxiety	Not Normal (Mild to Extremely Severe)	73/393 (18.6%)	47/218 (21.6%)	26/175 (14.9%)	0.09
	Normal	320/393 (81.4%)	171/218 (78.4%)	149/175 (85.1%)	
Stress	Not Normal (Mild to Extremely Severe)	97/393 (24.7%)	64/218 (29.4%)	33/175 (18.9%)	**0.016**
	Normal	296/393 (75.3%)	154/218 (70.6%)	142/175 (81.1%)	
Professional fulfillment (> = 3)	No	292/392 (74.5%)	163/218 (74.8%)	129/174 (74.1%)	0.89
	Yes	100/392 (25.5%)	55/218 (25.2%)	45/174 (25.9%)	
Burnout (mean workload, depersonalization > = 1.33)	No	233/393 (59.3%)	117/218 (53.7%)	116/175 (66.3%)	**0.011**
	Yes	160/393 (40.7%)	101/218 (46.3%)	59/175 (33.7%)	

All P-values were obtained from Chi-square tests or Fisher’s exact tests.

† Fisher’s exact test. For DASS-21: Depression (0–9 normal; 10–13 mild; 14–20 moderate;21–27 severe; 28+ extremely severe); Anxiety (0–7 normal; 8–9 mild; 10–14 moderate; 15-19severe; 20+ extremely severe); Stress (0–14 normal; 15–18 mild; 19–25 moderate; 26–33 severe; 34+ extremely severe).

Similarly, the exposed group had a higher prevalence of stress (29.4% vs. 18.9%, *P* = 0.016), and burnout (46.3% vs. 33.7%, *P* = 0.011); and marginally higher prevalence of anxiety (21.6% vs. 14.9%, *P* = 0.089), Both groups had similar prevalence of depression (28% vs. 26.3%, *P* = 0.70). Surprisingly, both groups had low professional fulfillment from their current clinical work activities (25.2% vs. 25.9%, *P* = 0.88) (see Table 1).

Finally, the overall usage of wellness resources by trainees was low. The use of institutional wellness programs was approximately 5%, though over 80% of the respondents were aware of the availability of these resources. Similarly, over 90% of the respondents were aware of the COVID-19 emergency resources such as housing, childcare and emotional support services, but only 4% had utilized these resources.

### Multivariable analysis

Multivariable model results indicated that trainees who were exposed to COVID-19 patients reported statistically significant higher stress (10.96 [95% CI, 9.65 to 12.46] vs 8.44 [95% CI, 7.3 to 9.76]; *P* = 0.043); the exposed group were also more likely to be burned out (1.31 [95% CI, 1.21 to1.41] vs 1.07 [95% CI, 0.96 to 1.19]; *P* = 0.002] (see Tables 2 and 3). There were no significant differences between the exposure groups for anxiety, depression or professional fulfillment (see S1–S3 Tables). We also found that female trainees were more likely to be stressed (*P* = 0.043) (see Table 3), whereas unmarried trainees were more likely to be depressed (*P* = 0.009), and marginally more likely to have anxiety (*P* = 0.051) (See S1 and S2 Tables).

**Table 2:** Univariable and multivariable negative binomial regression models for outcomes related to stress.

Variable	Group	Unadjusted mean (95% CI)	Univariable *P*-value	Adjusted mean (95% CI)	Multivariable *P*-value
Exposure to patients being tested for COVID-19	No	8.64 (7.48, 9.99)	0.017	8.44 (7.3, 9.76)	**0.011**
	Yes	10.96 (9.65, 12.46)		10.86 (9.56, 12.33)	
Clinical Role	Fellow	10.77 (9.13, 12.71)	0.225	-	-
	Resident	9.5 (8.44,10.7)		-	
Caucasian	No	9.58 (8.18,11.22)	0.578	-	-
	Yes	10.14 (8.97,11.45)		-	
Female	No	8.94 (7.73,10.33)	0.067	8.66 (7.49, 10.01)	**0.043**
	Yes	10.72 (9.43,12.2)		10.58 (9.31, 12.02)	
Children at home	No	9.68 (8.66, 10.82)	0.387	-	-
	Yes	10.67 (8.8, 12.94)		-	
Married*	No	9.87 (8.54,11.41)	0.921	-	-
	Yes	9.97 (8.76, 11.35)		-	
Year in program		0.998 (0.922,1.079)	0.956	-	-

Unadjusted means correspond to means and slope (year in program) unadjusted for covariates. Adjusted means correspond to means from multivariable models adjusted for those covariates that had *P* < 0.10 in univariable analyses. Negative binomial regression results are presented as back-transformed (inverse log link) means and slope (year in program).

**Table 3:** Univariable and multivariable linear least-squares regression models for outcomes related to burnout.

Variable	Group	Unadjusted mean (95% CI)	Univariable *P*-value	Adjusted mean (95% CI)	Multivariable *P*-value
Exposure to patients being tested for COVID-19	No	1.06 (0.95, 1.18)	0.002	1.07 (0.96, 1.19)	**0.0023**
	Yes	1.31 (1.21, 1.41)		1.31 (1.21, 1.41)	
Clinical Role	Fellow	1.11 (0.98, 1.25)	0.116	-	-
	Resident	1.24 (1.15, 1.34)		-	
Caucasian	No	1.17 (1.04, 1.3)	0.554	-	-
	Yes	1.22 (1.12, 1.31)		-	
Female	No	1.16 (1.04, 1.27)	0.337	-	-
	Yes	1.23 (1.13, 1.34)		-	
Children at home	No	1.22 (1.13, 1.31)	0.450	-	-
	Yes	1.15 (0.99, 1.3)		-	
Married*	No	1.27 (1.16, 1.39)	0.096	1.25 (1.13, 1.36)	0.15
	Yes	1.14 (1.04, 1.24)		1.14 (1.03, 1.24)	
Year in program		-0.043 (-0.11, 0.02)	0.16	0.157	-

Unadjusted means correspond to means and slope (year in program) unadjusted for covariates. Adjusted means correspond to means from multivariable models adjusted for those covariates that had P < 0.10 in univariable analyses.

## Discussion

Based on a cross-sectional survey of physician trainees, we found that trainees exposed to COVID-19 patients were significantly more stressed and experienced greater burnout. In addition, women trainees were more likely to have higher stress, and unmarried trainees were more likely to experience depression and anxiety. Compared to the prevalence of depression, anxiety and stress in the general population (12%, 11% and 11% respectively) [20], COVID-exposed trainees had higher prevalence rates (28%, 22%, and 29% respectively). Of these, the prevalence of depression among trainees is similar to those reported in the literature [12]; rates of stress and anxiety have not been previously reported for trainees in the United States. Burnout rates (41%) was similar to previously reported rates among residents [11]. Interestingly, the overall prevalence of burnout in the non-exposed group was lower (33%), which may be related to modifications of trainee schedules such as reduced work hours or remote work. The impact of the pandemic on proximal stressors such as childcare and work-family balance was also significantly higher among the exposed group, illustrating the multi-faceted stressors introduced by the pandemic.

This survey was conducted when the number of COVID-19 related hospitalizations was approximately 98 patients/day. The region is continuing to experience a “long tail” of COVID-19 related hospitalizations. It is likely that repeated and persistent exposure, will have considerable impact on the mental health and well-being of trainees over time. Additionally, new trainees, specifically in new regional hotspots, are still in high pressure situations where their altruistic goal of patient care maybe affected by the constant exposure to the pressures and stress associated with caring for COVID-19 patients [13, 24]. As such, strategies for mitigating the effects of chronic burnout and stress are urgent among this vulnerable group to prevent a “parallel pandemic,”[13] one potentially leading to loneliness, distress, substance abuse and other chronic clinical conditions [25, 26].

Although the true effects of this pandemic on trainees cannot be determined until long after the pandemic has ended, the findings from this survey highlight the proximal challenges that are currently faced by physicians in training. In a systematic review, De Brier et al. (2020) found that organizational and social support, clear communication, and developing a sense of control were protective factors in mitigating adverse mental health outcomes among healthcare workers during epidemics [5]. Several recent reports have suggested that immediate organizational support is needed in addressing the challenges faced by frontline healthcare workers including trainees. Support needed by frontline healthcare workers include basic needs (e.g. nutrition), availability of PPE, support for childcare, and institutional psychosocial and mental health support. These efforts should be coupled with focused and direct communication efforts to normalize discussion of distress, and resources to address wellness [27, 28]. The integration of wellness programs and initiatives into traditional “COVID-19 Command Centers,” the availability of anonymous psychological support resources, and most importantly, preventing the closure of existing wellness programs are considered critical for protecting the wellness of the healthcare workforce [13].

It is also important to highlight the low utilization of existing services by a group that clearly has a high prevalence of stress and burnout. The GME wellness office offers several programs including free psychiatric services, educational offerings for reducing burnout and stress, mindfulness training, and support programs such as childcare and housing services. There were also additional COVID-19 resources offering group support sessions. However, the low utilization of all of these resources is potentially related to the fact that trainees may feel reluctant to acknowledge their vulnerability to supervisors and peers.

To increase utilization of support services, it is necessary to normalize feelings of emotional distress and reduce stigma by encouraging discussion of the stressors of clinical work and high prevalence of mental distress and encouraging trainees (and their supervisors) to seek support when needed. The stigma associated with seeking care for mental health is particularly high among trainees; barriers related to cost, confidentiality, questions related to medical licensure and credentialing, and time, as well as the difficulty of finding a provider are significant [29, 30]. Programs that increase accessibility to mental health services for trainees by offering services that are centrally located on campuses and free of charge, along with education, prevention and outreach efforts are successful in supporting the mental health needs of trainees [31, 32].

Most experts in the field agree that interventions to address burnout should also include a focus on system-level issues [33, 34]. With serious economic concerns at academic medical centers due to canceled elective procedures and closed clinics [35], budgetary cuts are expected. Such budget cuts may introduce considerable challenges in addressing system-level concerns such as adding ancillary support to reduce non-physician tasks, interventions aimed at improving clinical workflows, or streamlining interactions with electronic health record systems. However, it is important that institutional leaders consider such budget cuts in the context of the economic impact of burnout [36], and the long-term impact on our health system from loss of our physician workforce. As emphasized by Dzau and colleagues in a recent editorial, investment in the clinician workforce is now more important than ever [13].

This study also highlights specific stressors including childcare and work-life balance, as well as the higher prevalence of stress for women, and higher prevalence of depression and burnout for unmarried trainees. Targeted interventions focusing on improving social-relatedness and connections have been shown to mitigate stress during training [37]. With mostly virtual interactions, programs must take extra effort to develop such social connections, especially among unmarried trainees. Similarly, with unpredictable schedules, night work and long hours, trainees often struggle to find adequate and affordable childcare [38]. Addressing and expanding the childcare options for trainees is, hence, paramount to reducing their overall stress. As the pandemic evolves, additional stressors related to missed educational opportunities and reduced job opportunities will also need to be evaluated and addressed.

Finally, in recent years, the Accreditation Council for Graduate Medical Education (ACGME) has added requirements for programs to address well-being in the learning and working environment, including a commitment to the well-being of students, residents, faculty and all members of the health care team. These new policies include requirements for programs to ensure trainees can attend medical appointments, have access to mental health services, attention to work schedules and minimization of non-physician tasks. Although this has been a big step in highlighting wellness needs, this mandate still lacks specificity in its requirements. Creating actionable requirements with specific outcome measures to evaluate the impact of wellness efforts will help in holding programs accountable for their efforts.

### Study limitations

This study has several limitations. This was a single academic medical center, cross-sectional study; associations between potential risk factors and considered outcomes should not be interpreted as causal. Planned longitudinal surveys will provide additional insights on the changes and long-term impact on trainee wellness. There is a potential response bias as participants who were distressed may not have completed the survey; in contrast, it is also possible that the distressed participants may have participated more as the topic of the survey was relevant to them. The response rate of 29%, although similar to response rate of other surveys among trainees [11], may not be representative all trainees. The survey did not capture other stress-related factors such as sleep, clinical workflow challenges or other emotional pressures that the trainees faced. This study also was conducted during the early stages of the COVID-19 pandemic in a region where physical distancing was enforced early, and the overall incidence was lower than other regions in the country. In spite of these limitations, the study represents the perspective of a large number of trainees (*n* = 393) whose experiences of stressors and burnout are likely to be experienced at other academic medical centers. These effects are likely to be more pronounced at infection hotspots.

## Conclusions

Physician trainees are among the healthcare workers who are at the forefront of care during COVID-19 pandemic. Their exposure to COVID-19 patients is associated with increased stress and burnout, with addition stressors arising from work-family concerns such as childcare. Given their complex role, as learners and frontline care providers, and their limited autonomy, they represent a vulnerable group, whose health and well-being during their formative years must be protected. Institutional, and more specifically, graduate medical education wellness programs, should focus on sustaining existing wellness programs and adding more accessible interventions that have a wider reach.

## Supporting information

S1 Table Multivariable model for anxiety using negative binomial regression. Unadjusted means correspond to means unadjusted for covariates. Adjusted means correspond to means multivariable model adjusted means that includes only those variables that had P < 0.10 in univariable analyses. Negative binomial regression results are presented as back-transformed (inverse log link) means and slope (year in program).
(DOCX)

S2 Table Multivariable model for depression using negative binomial regression. Unadjusted means correspond to means unadjusted for covariates. Adjusted means correspond to means multivariable model adjusted means that includes only those variables that had P < 0.10 in univariable analyses. Negative binomial regression results are presented as back-transformed (inverse log link) means and slope (year in program).
(DOCX)

S3 Table Multivariable model for professional fulfillment using least-square regression. Unadjusted means correspond to means unadjusted for covariates. Adjusted means correspond to means multivariable model adjusted means that includes only those variables that had P < 0.10 in univariable analyses.
(DOCX)
