# Supplementary material for: Exposure to COVID-19 patients increases physician trainee stress and burnout
Source: PLoS One. 2020 Aug 6;15(8):e0237301. doi: 10.1371/journal.pone.0237301 (PMC7410237; doi:10.1371/journal.pone.0237301)
Supplement: S2 Table — Unadjusted means correspond to means unadjusted for covariates. Adjusted means correspond to means multivariable model adjusted means that includes only those variables that had P < 0.10 in univariable analyses. Negative binomial regression results are presented as back-transformed (inverse log link) means and slope (year in program). (DOCX) [file pone.0237301.s002.docx]

**S2 Table**. Multivariable model for depression using negative binomial regression. Unadjusted means correspond to means unadjusted for covariates. Adjusted means correspond to means multivariable model adjusted means that includes only those variables that had P < 0.10 in univariable analyses. Negative binomial regression results are presented as back-transformed (inverse log link) means and slope (year in program).

| **Variable** | **Group** | **Unadjusted mean (95% CI)** | **Univariable P-value** | **Adjusted mean (95% CI)** | **Multivariable P-value** |  |
| --- | --- | --- | --- | --- | --- | --- |
| Exposure to patients being tested for COVID-19 | No | 6.55 (5.51, 7.78) | 0.71 | 6.69 (5.63, 7.95) | 0.9 |  |
|  |  |  |  |  |  |  |
| Clinical Role | Fellow | 7 (5.74, 8.53) | 0.610 | - | - |  |
|  | Resident | 6.57 (5.7, 7.56) |  | - |  |  |
| Caucasian | No | 7.06 (5.85, 8.52) | 0.5 | - | - |  |
|  |  |  |  |  |  |  |
| Female | No | 6.23 (5.24, 7.4) | 0.27 | - | - |  |
|  | Yes | 7.1 (6.09, 8.28) |  | - |  |  |
| Children at home | No | 6.87 (6.02, 7.84) | 0.49 | - | - |  |
|  |  | 6.24 (4.96, 7.87) |  | - |  |  |
| Married | No | 7.87 (6.65, 9.33) | 0.0082 | 7.86 (6.63, 9.33) | 0.0088 |  |
|  | Yes | 5.78 (4.95, 6.74) |  | 5.78 (4.95, 6.74) |  |  |
| Year in program |  | 1.024 (0.937,1.12) | 0.6 | - | - |  |
