# Supplementary material for: Exposure to COVID-19 patients increases physician trainee stress and burnout
Source: PLoS One. 2020 Aug 6;15(8):e0237301. doi: 10.1371/journal.pone.0237301 (PMC7410237; doi:10.1371/journal.pone.0237301)
Supplement: S3 Table — Unadjusted means correspond to means unadjusted for covariates. Adjusted means correspond to means multivariable model adjusted means that includes only those variables that had P < 0.10 in univariable analyses. (DOCX) [file pone.0237301.s003.docx]

**S3 Table**. Multivariable model for professional fulfillment using least-square regression. Unadjusted means correspond to means unadjusted for covariates. Adjusted means correspond to means multivariable model adjusted means that includes only those variables that had P < 0.10 in univariable analyses.

| **Variable** | **Group** | **Unadjusted mean (95% CI)** | **Univariable P-value** | **Adjusted mean (95% CI)** | **Multivariable P-value** |  |
| --- | --- | --- | --- | --- | --- | --- |
| Exposure to patients being tested for COVID-19 | No | 2.2 (2.06, 2.33) | 0.66 | 2.22 (2.08, 2.36) | 0.61 |  |
|  |  |  |  |  |  |  |
| Clinical Role | Fellow | 2.33 (2.18, 2.49) | 0.085 | 2.32 (2.17, 2.48) | 0.1 |  |
|  | Resident | 2.16 (2.05, 2.27) |  | 2.16 (2.05, 2.28) |  |  |
| Caucasian | No | 2.17 (2.02, 2.32) | 0.42 | - | - |  |
|  |  |  |  |  |  |  |
| Female | No | 2.31 (2.17, 2.44) | 0.09 | 2.32 (2.18, 2.46) | 0.095 |  |
|  | Yes | 2.15 (2.03, 2.27) |  | 2.17 (2.04, 2.29) |  |  |
| Children at home | No | 2.2 (2.1, 2.31) | 0.56 | - | - |  |
|  |  |  |  |  |  |  |
| Married | No | 2.13 (2, 2.27) | 0.090 | 2.17 (2.03, 2.32) | 0.12 |  |
|  | Yes | 2.29 (2.17, 2.41) |  | 2.32 (2.19, 2.44) |  |  |
| Year in program |  | 0.027 (-0.045, 0.098) | 0.46 | - | - |  |
